# Review of Breast Cancer Pathologigcal Image Processing

**DOI:** 10.1155/2021/1994764

**Published:** 2021-09-20

**Authors:** Ya-nan Zhang, Ke-rui XIA, Chang-yi LI, Ben-li WEI, Bing Zhang

**Affiliations:** ^1^School of Computer Science and Technology, Harbin University of Science and Technology, Harbin 150080, China; ^2^HRG International Institute (Hefei) of Research and Innovation, Hefei 230000, China

## Abstract

Breast cancer is one of the most common malignancies. Pathological image processing of breast has become an important means for early diagnosis of breast cancer. Using medical image processing to assist doctors to detect potential breast cancer as early as possible has always been a hot topic in the field of medical image diagnosis. In this paper, a breast cancer recognition method based on image processing is systematically expounded from four aspects: breast cancer detection, image segmentation, image registration, and image fusion. The achievements and application scope of supervised learning, unsupervised learning, deep learning, CNN, and so on in breast cancer examination are expounded. The prospect of unsupervised learning and transfer learning for breast cancer diagnosis is prospected. Finally, the privacy protection of breast cancer patients is put forward.

## 1. Introduction

Breast cancer is one of the most common malignant tumors. According to Chinese Women's survey, breast cancer is the most common malignant tumor in Chinese women, and the incidence rate is increasing year by year. The key to reduce the mortality of breast cancer is early diagnosis and treatment. At present, mammography is the most commonly used method to detect breast cancer. However, because of the huge amount of data and the poor imaging features of early breast cancer, early diagnosis is very difficult. With the development of image processing technology and early diagnosis technology, image processing of breast pathology has become an important way of early diagnosis of breast cancer, which mainly includes the study of masses, calcifications, and breast density. One of the main manifestations of breast cancer in breast mammography is mass. The basic steps of pathological image processing are as follows: first is image preprocessing, which includes removing background, marker, pectoral muscle and noise, and breast segmentation and image enhancement. Secondly, the region of interest is found by a basic image processing method. Then, features that can represent the quality, such as texture features and morphological features, are extracted. Finally, the tumor and normal tissue were separated according to the extracted features. Another manifestation of breast cancer on X-ray images is a large breast density [1].

The object of breast pathology image processing is a variety of medical images with different imaging mechanisms. The types of medical imaging widely used in clinic include X-ray imaging (X-CT), magnetic resonance imaging (MRI), nuclear medicine imaging (NMI), and ultrasonic imaging (UI). X-ray imaging (X-CT), mainly through X-ray tomography, such as head tomography, is used to diagnose cerebral vascular diseases and intracranial hemorrhage. X-ray tomography has a good effect in the diagnosis of traumatic skull and facial fractures. Magnetic resonance imaging (MRI) is a noninvasive imaging technique, which can produce three-dimensional anatomical images [2]. Nuclear medicine imaging (NMI) is based on the difference of radioactivity concentration inside and outside organs or between different parts of organs [3, 4]. UI was used to observe the shape, location, size, number and scope of the mass, and the activity of abdominal organs. The edge echo, capsule, smoothness, wall thickness, and halo were observed. According to the clinical data, the symptoms of breast cancer can be adjusted by means of X-ray examination, which is identified by X-ray mammography. MRI results of tumor detection are higher than the actual value, while CT results of tumor detection are lower than the actual value. Dynamic enhanced CT examination of breast lesions can better overcome the situation of missed detection.

Medical image acquisition and interpretation are the basis of breast cancer diagnosis based on medical imaging. In recent years, the speed and resolution of image acquisition have been greatly improved. However, image diagnosis is limited by the doctor's experience, ability, and other subjective factors, and its ability of replication and promotion is limited. In order to minimize the dependence on doctors, image processing technology is applied to medical imaging processing. Medical image processing includes lesion detection, image segmentation, image registration, and image fusion. In addition to clinical diagnosis, medical image processing also plays an important auxiliary role in medical teaching, operation planning, operation simulation, and various medical researches [5, 6].

## 2. Detection of Breast Cancer

Detection of breast cancer is mainly based on detection methods, image processing of lesion detection, lesion location matching, and extraction of lesion feature values. Breast cancer detection can detect candidate lesion location by supervised learning or classical image processing. The combination of image processing and Convolutional Neural Networks (CNN) is one of the successful examples of deep learning in recent years. The CNN is applied in breast cancer image analysis, mapping the input layer, pool layer, modified linear unit, all link layer, and output layer, respectively, and predicting the information represented by medical images. For example, Setio et al. extracted the features of pulmonary nodules in nine different directions of 3D chest CT scanning, selected the appropriate candidate as the center, and classified the candidates through CNN [3, 7–11]. Ross et al. decomposed the 3D image into 2D patches and then rotated the 2D patches randomly to get the “2.5D” view. The CNN was used to detect the early features of cancer from the 2.5D view. The combination of deep learning and image processing greatly improves the accuracy of lesion detection [12], while it is difficult to achieve high accuracy by using nondeep learning classifiers such as support vector machine. The accuracy of CNN algorithm depends on the training of initial markers by experts and needs a wide range of case coverage. Therefore, the promotion of CNN in the field of medical image processing is constrained by resources “transfer learning” that can reduce the dependence of CNN on initial marker training to a certain extent [13, 14], but the application of transfer learning itself is limited, so it is difficult to find the application conditions of transfer learning between medical images of human organs.

Early diagnosis of breast cancer can be detected by tumor markers. Tumor markers are substances produced and secreted by tumor cells during growth and reproduction. When these substances reach a certain amount, they can be extracted from breast images. The early feature values of breast cancer can be identified by using SIFT (scale invariant feature transform) or HOG (Histogram of Oriented Gradient) and so on to provide support for the early diagnosis of breast cancer. The combination of image processing technology and reinforcement learning technology can reduce the dependence on a human doctor's experience to the greatest extent. Image processing technology is used to process two-dimensional slices, and then reinforcement learning is combined to set the enhancement target. Through the judgment of each discrete two-dimensional slice, the optimal decision strategy is found, so as to maximize the benefit of judging the pathological correctness of the whole group of two-dimensional slices. Through the analysis and processing of two-dimensional slice image, the segmentation, extraction, three-dimensional reconstruction, and three-dimensional display of human breast, surrounding soft tissue and lesion are realized. After the calibration of features, the reinforcement learning is used to quantitatively analyze the lesion and the region around the breast. Combined with the revenue target, the learning is carried out through continuous attempts. The goal is to obtain the maximum revenue value. Based on the reinforcement learning method, it is found that breast cancer does not need to know how to produce correct breast cancer recognition action. Reinforcement learning relies on its own learning, constantly trying and making mistakes and constantly recording the maximum value of income in the process of trial and error until the method of finding the maximum value of income is found.

## 3. Breast Cancer Image Segmentation

Based on the given feature factors, the medical image segmentation compares the similarity of feature factors between images and divides the image into several regions. The objects of medical image segmentation mainly include cells, tissues, and organs. The region-based segmentation method is mainly based on the spatial local features of the image, such as gray, texture, and other pixel statistical characteristics. The boundary-based segmentation method mainly uses gradient information to determine the boundary of the target. For example, the fast marching algorithm and the medical image segmentation method of watered transform can segment the image quickly and accurately [15].

In recent years, with the development of other emerging disciplines, image segmentation technology develops rapidly, and new methods generated by interdisciplinary emerge in endlessly. Some new image segmentation techniques have been developed for breast cancer detection, such as the method based on statistics, the method based on fuzzy theory, the method based on neural network, the method based on wavelet analysis, the model-based snake model (dynamic contour model), and the combination optimization model. Although new segmentation methods have been proposed, the results are not ideal. At present, the research focus is a knowledge-based segmentation method; that is, some prior knowledge is introduced into the segmentation process by some means, so as to constrain the computer segmentation process, so that the segmentation results can be controlled within the range we can understand without going too far [16]. For example, when the gray value of the tumor in the liver is very different from that of the normal liver, the tumor and the normal liver will not be regarded as two independent tissues. All of the above methods have their own limitations. The results are good in specific scenarios, but not ideal beyond specific scenarios. Because the boundaries of internal organs, muscles, blood vessels, and other organs are usually very complex, the diseased areas of organs are mixed with normal tissues, and the differences between the gray levels and boundaries of diseased areas and normal tissues are relatively small, it is very difficult to identify these organs and diseased areas in medical images, and the existing image segmentation algorithms can not complete the task of image segmentation independently. Human intervention is also needed [17]. Medical image segmentation is quite different from image segmentation in other fields. The effect of existing classical algorithms in medical image segmentation is not good. It is still necessary to continue to study in improving the accuracy, speed, adaptability, and robustness of image segmentation [18]. The image segmentation method based on prior knowledge can well control the segmentation boundary of the image. For example, in the segmentation of intrahepatic mass, the image segmentation method based on prior knowledge can recognize intrahepatic mass and normal liver by gray value. However, image segmentation based on prior knowledge requires a large number of prior data. The more prior data, the more accurate the results. For example, Ghesu et al., based on 2891 times of cardiac ultrasound data, used deep learning and edge space learning to detect and segment cardiac ultrasound images [17, 18]. Parameter space exploration and data sparsity are important factors to improve the efficiency of medical image segmentation. Brosch et al. proposed a 3D deep convolution coder network through convolution and deconvolution to segment multiple sclerosis brain lesions and normal brain regions [19]. Data normalization and data enhancement techniques are applied to image enhancement and core regions of suspected tumors in brain tumor segmentation research and achieved good results [20]. The research of medical image segmentation methods has the following remarkable characteristics: it is difficult for any single existing image segmentation algorithm to achieve satisfactory results for general images, so more attention should be paid to the effective combination of multiple segmentation algorithms. Due to the complexity of human anatomical structure and the systematicness of function, although there have been studies on the methods of automatic segmentation of medical images to distinguish the required organs and tissues or find the lesion area, the existing software packages generally can not complete the automatic segmentation, and the manual intervention of anatomy is still needed [21]. At present, it is impossible for computer to complete the task of image segmentation, so the human-computer interactive segmentation method has gradually become the focus of research. The research of new segmentation methods mainly focuses on automatic, accurate, fast, adaptive, and robust features. The comprehensive utilization of classical segmentation technology and modern segmentation technology is the development direction of medical image segmentation technology in the future [22, 23].

## 4. Breast Cancer Image Registration

Image registration is the first mock exam of image fusion. In breast cancer clinical diagnosis, multiple modes or modes of image registration and fusion are needed. More information can help doctors give more accurate diagnosis [24]. In the clinical diagnosis process of breast cancer, medical image registration mainly locates reference points in two or more images; through spatial location transformation, such as rotation, the reference point is located in a coordinate system. Registration requires that the mapping of points between images is one-to-one correspondence; that is, each point in an image space has corresponding points in another image space, or in the sense of medical diagnosis, the points in the image can be accurately or approximately accurately corresponded [25–27]. Registration can be divided into two types: based on external features and based on internal features. Registration based on image internal features is noninvasive and traceable, which is the focus of research on registration algorithm [28].

There are two major categories of medical registration research for breast cancer: (1) a deep learning network is used to estimate the similarity of two images and drive iterative optimization, and (2) the depth regression network is directly used to predict the conversion parameters. The former only uses deep learning for similarity measurement and still needs the traditional registration method for iterative optimization. It does not give full play to the advantages of deep learning, takes a long time, and is difficult to achieve real-time registration. Therefore, only the latter is studied and discussed, and the conclusion is limited to this kind of nonrigid registration method. Based on supervised learning, there are two ways to obtain tags: (1) the traditional classical registration method is used for registration, and the deformation field is used as tags. (2) The original image is simulated as a fixed image, the deformed image as a moving image, and the simulated deformation field as a label. Based on unsupervised learning, the registration pair is input into the network to obtain the deformation field, and the moving image is interpolated to obtain the registration image. The 3D image is similar to it. The 3D image is input into the network to obtain the deformation field (dx, dy, dz), and then, the registration image is obtained by interpolation. However, the medical image registration of breast cancer is still an unsolved classic problem. There are no universally recognized gold standard and no corresponding large database in this field. Deep learning methods have some successful cases in breast cancer image registration. There are usually several reasons: (1) the expert knowledge of the field is well utilized, (2) the data are properly preprocessed and processed by data enhancement, (3) a special network structure is designed for a single task, and (4) the appropriate super parameter optimization method is used: such as parameter adjustment based on intuition or the Bayesian method. There are still some difficulties and challenges in the field of breast cancer medical image registration: (1) there is a lack of large databases with precise annotation. (2) Specific tasks need guidance from experts in the field. (3) It is difficult to agree with the opinions of different experts in some ambiguous images. (4) The two classification models are too simple to be competent for more complex cases. (5) the difficulties of breast cancer medical image analysis still exist in images. Besides the analysis, we need to make full use of the information about other dimensions of the patient, like age, medical history, etc. (6) The slice-based neural network is having difficulty in using the location information of the corresponding anatomical structure in the original image, but the method of transferring the whole image into the neural network has corresponding disadvantages. Although Esteva et al. made amazing progress in dermatology in 2017 and Gulshan et al. in ophthalmology in 2016, they have achieved image classification models with higher accuracy than human experts in both fields. However, the essential reason for its success is that the above two problems are simple enough, and the data volume of ImageNet dataset is very large, so the existing model is applied to the above two problems and has achieved very good results. Usually, there is no such simple structure, and there is no effective network structure that can be used for 3D gray or multichannel image preprocessing. The advantage of unsupervised learning over supervised learning is that unsupervised learning does not need a large number of precisely labeled data. In addition, unsupervised learning imitates the way of human learning and can automatically perform the required tasks without special labeling or can deal with a large number of classification problems with little supervision. At present, the main methods of unsupervised learning are self-coding VAE and counter neural network Gan. Compared with accurately labeled breast cancer medical images, unlabeled breast cancer medical image data are easier to obtain. Unsupervised learning can directly use standard breast cancer medical images without any supervision, so it has more advantages. Finally, because deep learning is similar to black box model, the interpretation of breast cancer medical image domain requires a higher model, and further work is needed. At present, some work includes introducing Bayesian statistics into deep learning, which will help to measure the uncertainty of prediction.

**Figure 1:**
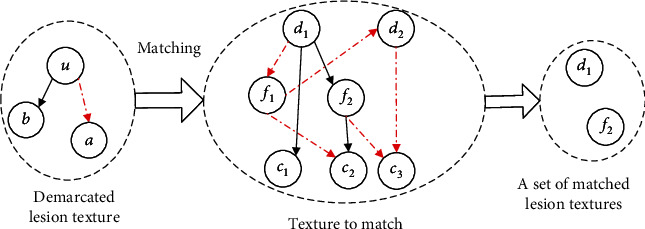
Image recognition sketch based on topological structure.

Multimodality medical image registration is a new direction of breast cancer registration, such as nonrigid multimodal medical image registration based on structure representation of PCANet [29]. PCANet can automatically learn intrinsic features from a large number of medical images through multilevel linear and nonlinear transformation, which has better information entropy than the artificial feature extraction method. Multilevel image features extracted from each layer of PCANet can effectively represent multimodal images. The fusion of medical image registration technology and informatics theory opens a new idea for medical image registration of breast cancer. For example, the principle of maximum information entropy is applied to image registration, which can maximize the diversity of information and retain the main information without neglecting the secondary information [30–35]. Three-dimensional multimode image registration is a new direction of medical image registration. It has more information than two-dimensional image and can support a doctor's diagnosis more effectively. In addition, some new image registration algorithms, such as image recognition of breast cancer based on topology, feature points are extracted from existing breast cancer images. They are combined into a matching area with a certain topological structure as the matching template. In the breast images to be matched, regions with similar topological structures are found; these regions may be breast cancer. The main steps of image recognition of breast cancer based on topology are as follows: (1) the first one is extracting feature points or feature regions of a specific scale and combining them into topological templates. (2) The topology of the image to be matched is extracted. (3) By comparing the topology in the image to be matched with the topology template, regions with similar topology are found. (4) The similarity between similar topology and feature points in topology template is compared, and the product of topology similarity and feature point similarity is regarded as the final similarity. The schematic diagram of image recognition based on topology structure is shown in Figure 1. In Figure 1, the left dotted line is the topology template, the middle dotted line is the topology extracted from the image to be matched, and the right dotted line is the region with similar topology [36].

Other methods such as algorithms based on wavelet transform, statistical parametric mapping algorithm, and genetic algorithm are also continuously integrated into breast cancer image registration. The combination of multiobjective optimization, reinforcement learning, and other methods with medical image registration is the future development direction of medical image registration.

## 5. Breast Cancer Image Fusion

Breast cancer image fusion extracts useful information from multiple images, filters redundant information, and improves the medical value of images. In general, image fusion from low to high is signal level fusion, data level fusion, feature level fusion, and decision level fusion.

1. Signal level: at the lowest level, the unprocessed sensor output is mixed in the signal domain to produce a fused signal. The fused signal has the same form as the source signal, but its quality is better. The signal from the sensor can be modeled as random variables mixed with different correlated noises. In this case, fusion can be considered an estimation process, and signal level image fusion is the optimal concentration or distribution detection problem of signal to a large extent, which requires the highest registration in time and space.
2. Pixel level: pixel level image fusion is the most basic fusion of the three levels. After pixel level image fusion, the obtained image has more detailed information, such as edge and texture extraction, which is conducive to the further analysis, processing, and understanding of the image. It can also expose the potential target, which is conducive to the operation of judging and identifying the potential target pixels. This method can save as much information as possible in the source image and increase the content and details of the fused image [37]. This advantage is unique and only exists in pixel level fusion. However, the limitations of pixel level image fusion can not be ignored, because it is to operate on pixels, so the computer has to process a large number of data, and the processing time will be relatively long, so the fused image can not be displayed in time and real-time processing can not be realized. In addition, in data communication, the amount of information is large, and it is easy to be affected by noise. In addition, if you directly participate in image fusion without strict image registration, the fused image will be blurred, and the target and details are not clear and accurate.
3. Feature level: feature level image fusion is to extract the feature information from the source image. The feature information is the information of the target or the region of interest in the source image, such as edge, person, building, or vehicle. Then, the feature information is analyzed, processed, and integrated to get the fused image features. The accuracy of target recognition based on fused features is obviously higher than that of the original image. The image information is compressed by feature level fusion and then analyzed and processed by computer. Compared with pixel level, the memory and time consumed will be reduced, and the real-time performance of the required image will be improved. Feature level image fusion requires less accuracy of image matching than the first layer, and its computing speed is faster than the first layer. However, it extracts image features as fusion information, so it will lose a lot of detail features.
4. Decision level: decision level image fusion is a cognitive-based method, which is not only the highest level of image fusion method but also the highest level of abstraction. Decision level image fusion is targeted [38]. According to the specific requirements of the problem, the feature information obtained from the feature level image is used, and then, the optimal decision is made directly according to certain criteria and the credibility of each decision, that is, the probability of the existence of the target. Among the three fusion levels, the calculation of decision level image fusion is the smallest, but this method has a strong dependence on the previous level, and the image is not very clear compared with the former two fusion methods. It is difficult to realize the decision level image fusion, but the noise has the least influence on the image transmission.

To sum up, data level fusion is the process of directly processing the collected data to obtain the fused image, which is the basis of high-level image fusion. Feature level fusion preserves the information contained in different images. Decision level fusion is the highest level of image fusion based on subjective needs. In breast medical image fusion, data level fusion is the main method. For example, multimodality medical image fusion is a technology that integrates multiple dimensions information. It can provide more comprehensive and accurate information for clinical detection of breast cancer [39]. The steps of image fusion are mainly divided into image data fusion and fusion image display. At present, the data fusion of breast image is mainly based on pixels, which process the image point by point and sum the gray values of the corresponding pixels of the two images. However, the image will be blurred to a certain extent after using this method. The fusion method based on breast image features needs to extract image features and do target segmentation and other processing on the image. The display of fusion image includes pseudocolor display, tomographic display, and three-dimensional display. Pseudocolor display takes an image as a benchmark and superimposes the gray and contrast features of the image to be fused with the benchmark image. The tomographic display method can display the fused three-dimensional data synchronously in cross-sectional, coronal, and sagittal images, which are convenient for the observer to diagnose. The three-dimensional display method, namely, three-dimensional reconstruction, is to display the fused breast data in the form of three-dimensional images, which can more intuitively observe the spatial anatomical position of the lesions. The earliest method of 3D reconstruction is back projection. At present, there are two common reconstruction methods: filtered back projection and convolution back projection. The information content of three-dimensional image is large, and the future three-dimensional image fusion technology will be a focus of image fusion research. With the development of interdisciplinary research, new image fusion methods are emerging. The application of wavelet transform, nonlinear registration based on finite element analysis, and artificial intelligence technology in breast image fusion will be the focus of image fusion research.

## 6. Forecast and Challenge

1. The application of unsupervised and supervised learning in the field of breast cancer image processing: breast cancer image classification based on CNN is the mainstream classification method nowadays. Fine tuning of CNN parameters directly affects the final image processing results. Unsupervised learning will have more promising results in breast cancer image processing
2. Transfer learning and fine tuning in the application of breast cancer image processing: transfer learning can partially alleviate the plight of not enough annotation data. In the process of transfer learning, a better plan is to use pretrained CNN as initialization of network and then carry out further supervision training. The annotated dataset is still a challenge in the field of breast cancer image processing
3. Patients pay more and more attention to privacy protection. The privacy of breast cancer image data has attracted much attention. In the process of breast cancer image processing, it is necessary to improve the accuracy of recognition, help doctors to give diagnosis, and pay attention to the protection of patients' privacy, protect the original information of the image from unauthorized access, and diagnose the whole process of image information authorized access and make the image access trace checkable

## 7. Conclusion

Deep learning and reinforcement learning are the relatively close combination of machine learning algorithm and breast cancer image processing and have made considerable progress. There are obvious differences between breast cancer image and other fields such as noise reduction, grayscale transformation, target segmentation, and feature extraction. The traditional image processing method is not directly applied to breast cancer image processing. Accumulation of deep learning in image processing can not be directly transferred to breast cancer image processing. Reinforcement learning belongs to unsupervised learning, which is different from deep learning. It uses an incentive mechanism that does not need a large number of sample space. Compared with deep learning, reinforcement learning has a wider applicability and lower promotion cost. Moreover, reinforcement learning has achieved very good results in chess, man-machine game, and other fields, which is suitable for complex logic processing. The combination of reinforcement learning and medical image processing will play a greater role in the clinical detection and prediction of breast cancer.
